# Pre-clinical alterations in cardiovascular phenotypes and their associations with metabolic profiles among obese youths

**DOI:** 10.1186/1687-9856-2015-S1-O44

**Published:** 2015-04-28

**Authors:** Elim Man, Yiu-Fai Cheung, Pik-To Cheung

**Affiliations:** 1 Department of Paediatrics & Adolescent Medicine, Queen Mary Hospital, the University of Hong Kong, Hong Kong SAR, China

## Introduction

Childhood obesity and various obesity-related co-morbidities have become a serious global healthcare problem. Predictive risk stratification and early detection of high-risk obese youths can potentially help modify the outcomes of obesity before irreversible damage taking place. Newer surrogate markers for assessing pre-clinical cardiovascular alterations have good predictive values for future cardiovascular risks. However, local data regarding obesity-related arterial alterations in youth are lacking at present.

## Aims

To evaluate the 1) pre-clinical cardiovascular alterations; and 2) their associations with the metabolic profiles in obese Hong Kong Chinese youths.

## Study methods

A total of 56 obese subjects and 58 healthy lean controls (M: F 1:1, age 17.0 ± 2.06 years) were recruited. Clinical, biochemical and arterial (structural and functional) parameters were obtained from all participants. Structural arterial parameters were determined by assessing carotid arterial intima-media thickness with ECG-synchronized ultrasound (Vivid 7, GE Medical Systems, Norway). Cross-sectional and regional arterial functions were determined by assessing the arterial stiffness (β) of carotid arteries and pulse wave velocities of various arterial segments respectively with an automatic device (VP-2000; Colin Medical Technology, Japan). All ultrasonographic and arterial assessments were performed by a single researcher.

## Results

Obese youths have worse preclinical arterial phenotypes both structurally and functionally, hence higher risks of developing cardiovascular disease in the future (p<0.0001). Important independent risk factors for preclinical arterial alterations in obese youths, including body mass indices (BMI), waist circumferences, blood pressures and plasma alanine aminotransferase (ALT) levels were demonstrated (p<0.0001). Obese youths having co-morbid metabolic disturbances were associated with worse preclinical arterial phenotypes. The worst cardiovascular phenotypes were shown in obese youths with the highest triglycerides to high-density lipoproteins (Tg-to-HDL) ratios despite most of them having normal serum triglyceride and HDL levels. Furthermore, obese subjects with higher paediatric NAFLD fibrosis index (PNFI) scores [1] have worse cross-sectional arterial stiffness.

## Conclusions

Assessment of preclinical cardiovascular phenotypes in asymptomatic obese youths provides clinicians a window for early identification of those at higher risks of developing future cardiovascular events. Evaluation of lipid compositions and obesity-related liver alterations may potentially help further stratify obese youths into subgroups with different degrees of cardiovascular risks.
